# Supplementary material for: Anti-inflammatory activity of the dietary supplement Houttuynia cordata fermentation product in RAW264.7 cells and Wistar rats
Source: PLoS One. 2020 Mar 25;15(3):e0230645. doi: 10.1371/journal.pone.0230645 (PMC7094840; doi:10.1371/journal.pone.0230645)
Supplement: S1 Table — (DOCX) [file pone.0230645.s001.docx]

**Supporting information**

**S1 Table Primer list**

| **Primers** | **Sequences** |
| --- | --- |
| COX-2-Sense | 5’-CCC-AGA-GCT-TTT-CAA-CC-3’ |
| COX-2-Antisense | 5’-ATT-TGG-CAC-ATT-TCT-TCC-CC-3, |
| iNOS-Sense | 5’-CCC-TTC-CGA-AGT-TTC-TGG-CAG-CAG-C-3’ |
| iNOS-Antisense | 5’-GGC-TGT-CAG-AGC-CTC-GTG-GCT-TTG-G-3’ |
| TNF-α-Sense | 5’-AGC-ACA-GAA-AGC-ATG-ATC-CG-3’ |
| TNF-α- Antisense | 5’-GTT-TGC-TAC-GAC-GTG-GGC-TA-3’ |
| IL-6-Sense | 5’-CGA-TGA-TGC-ACT-TGC-AGA-AA-3, |
| IL-6- Antisense | 5’-TGG-AAA-TTG-GGG-TAG-GAA-GG-3’ |
| IL-1β-Sense | 5’-TGC-AGA-GTT-CCC-CAA-CTG-GTA-CAT-C-3’ |
| IL-1β-Antisense | 5’-GTG-CTG-CCT-AAT-GTC-CCC-TTG-AAT-C -3’ |
| β-Actin-Sense | 5’-TCA-TGA-AGT-GTG-ACG-TTG-ACA-TCC-GT-3’ |
| β-Actin-Antisense | 5’-CCT-AGA-AGC-ATT-TGC-GGT-GCA-CGA-TG-3, |
