# Supplementary material for: Anti-inflammatory activity of the dietary supplement Houttuynia cordata fermentation product in RAW264.7 cells and Wistar rats
Source: PLoS One. 2020 Mar 25;15(3):e0230645. doi: 10.1371/journal.pone.0230645 (PMC7094840; doi:10.1371/journal.pone.0230645)

The images of mRNA expression were captured by Gel Documentation and analyzed by automated imaging (Bio Rad).

iNOS mRNA expression

HCFP aqueous extract

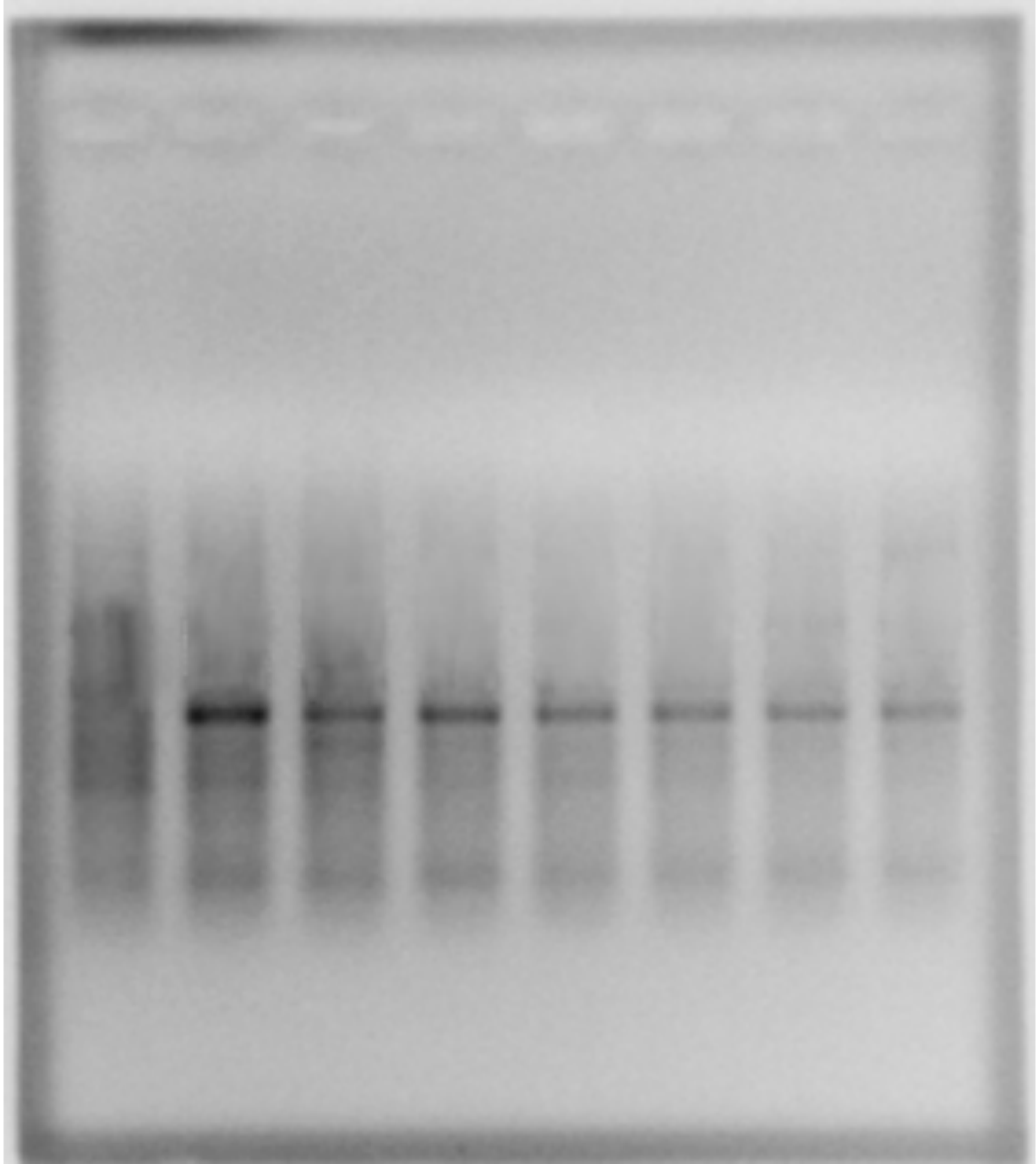

HCFP aqueous extract (µg/mL)	-	-	-	25	50	250	550	750
LPS (1 µg/mL)	-	+	+	+	+	+	+	+
DCF (25 µg/mL)	-	-	+	-	-	-	-	-

HCFP methanolic extract

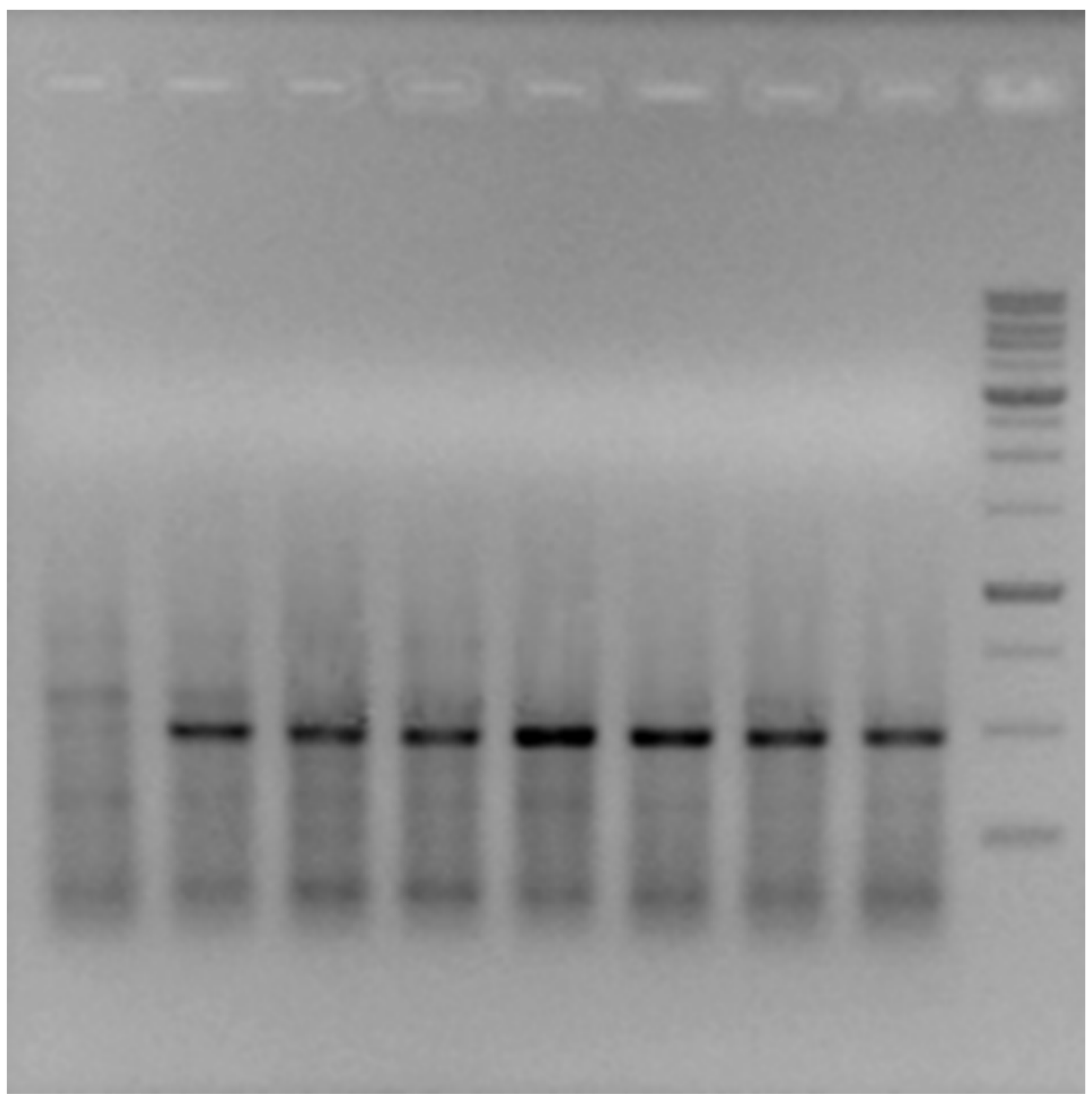

HCFP methanolic extract (µg/mL)	-	-	-	4	6	8	10	12
LPS (1 µg/mL)	-	+	+	+	+	+	+	+
DCF (25 µg/mL)	-	-	+	-	-	-	-	-

COX-2 mRNA expression

HCFP aqueous extract HCFP methanolic extract

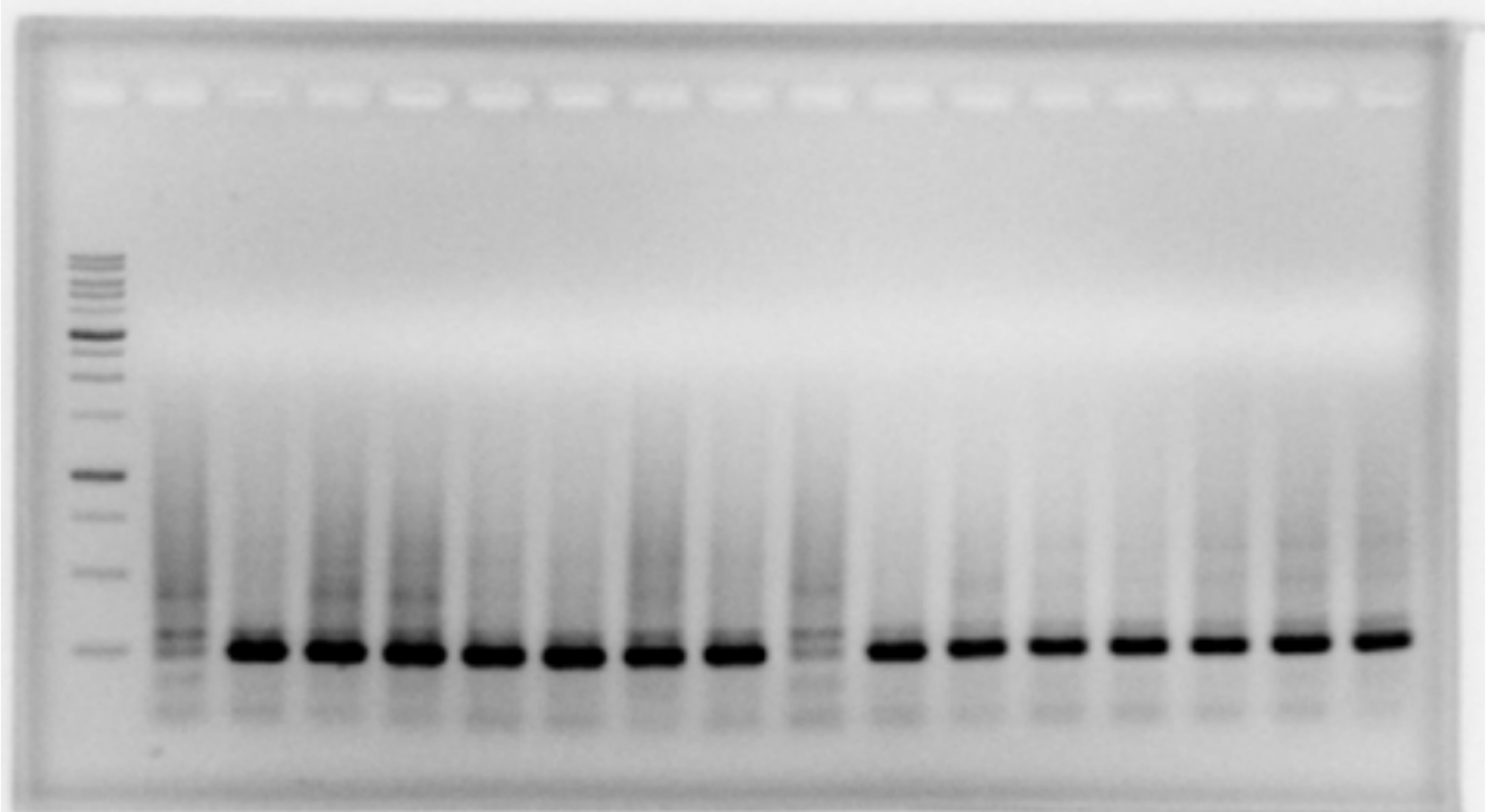

HCFP aqueous extract (µg/mL)	-	-	-	25	50	250	550	750	-	-	-	4	6	8	10	12	HCFP methanolic extract (µg/mL)
LPS (1 µg/mL)	-	+	+	+	+	+	+	+	-	+	+	+	+	+	+	+	LPS (1 µg/mL)
DCF (25 µg/mL)	-	-	+	-	-	-	-	-	-	-	+	-	-	-	-	-	DCF (25 µg/mL)

IL-1β mRNA expression

HCFP aqueous extract

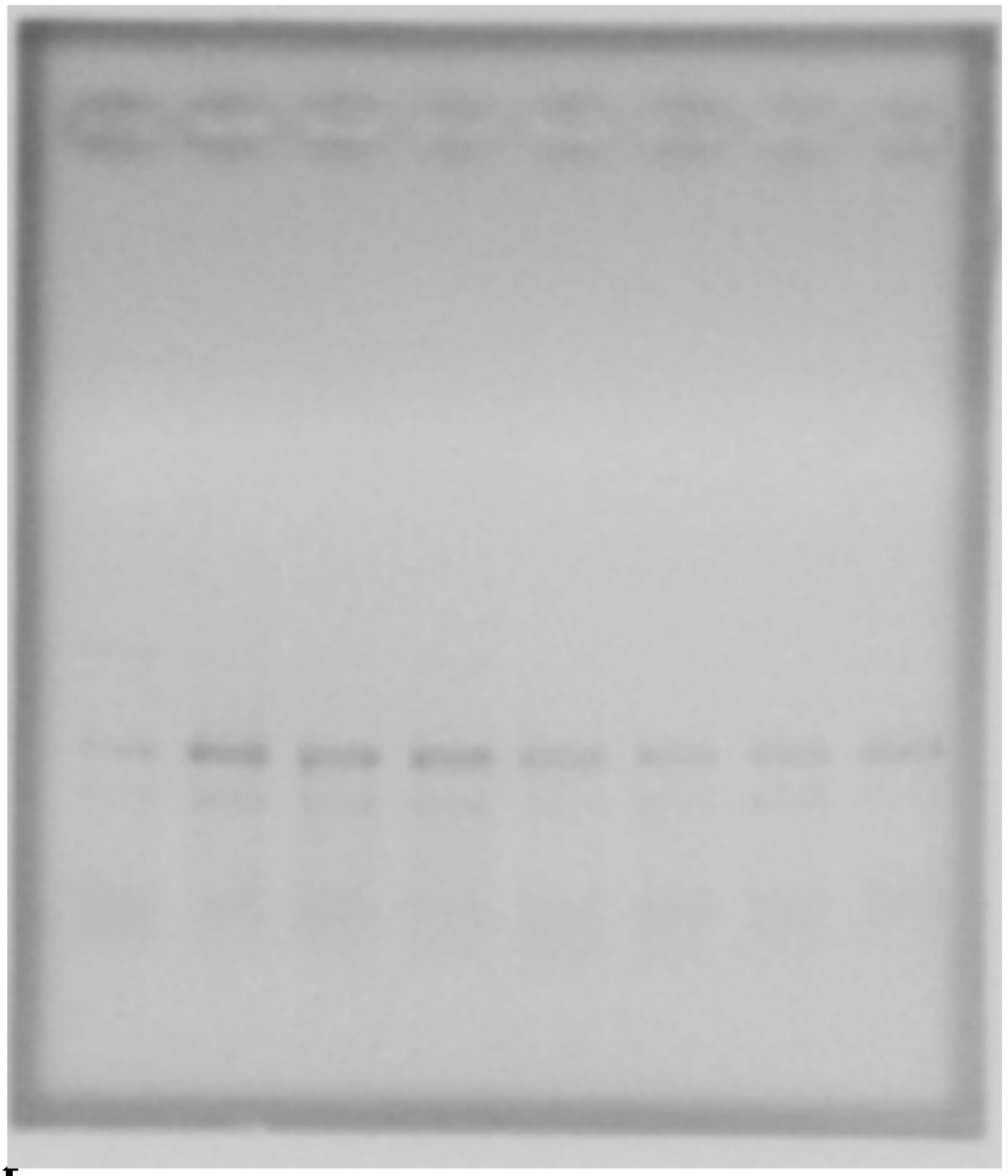

HCFP aqueous extract (µg/mL)	-	-	-	25	50	250	550	750
LPS (1 µg/mL)	-	+	+	+	+	+	+	+
DCF (25 µg/mL)	-	-	+	-	-	-	-	-

HCFP methanolic extract

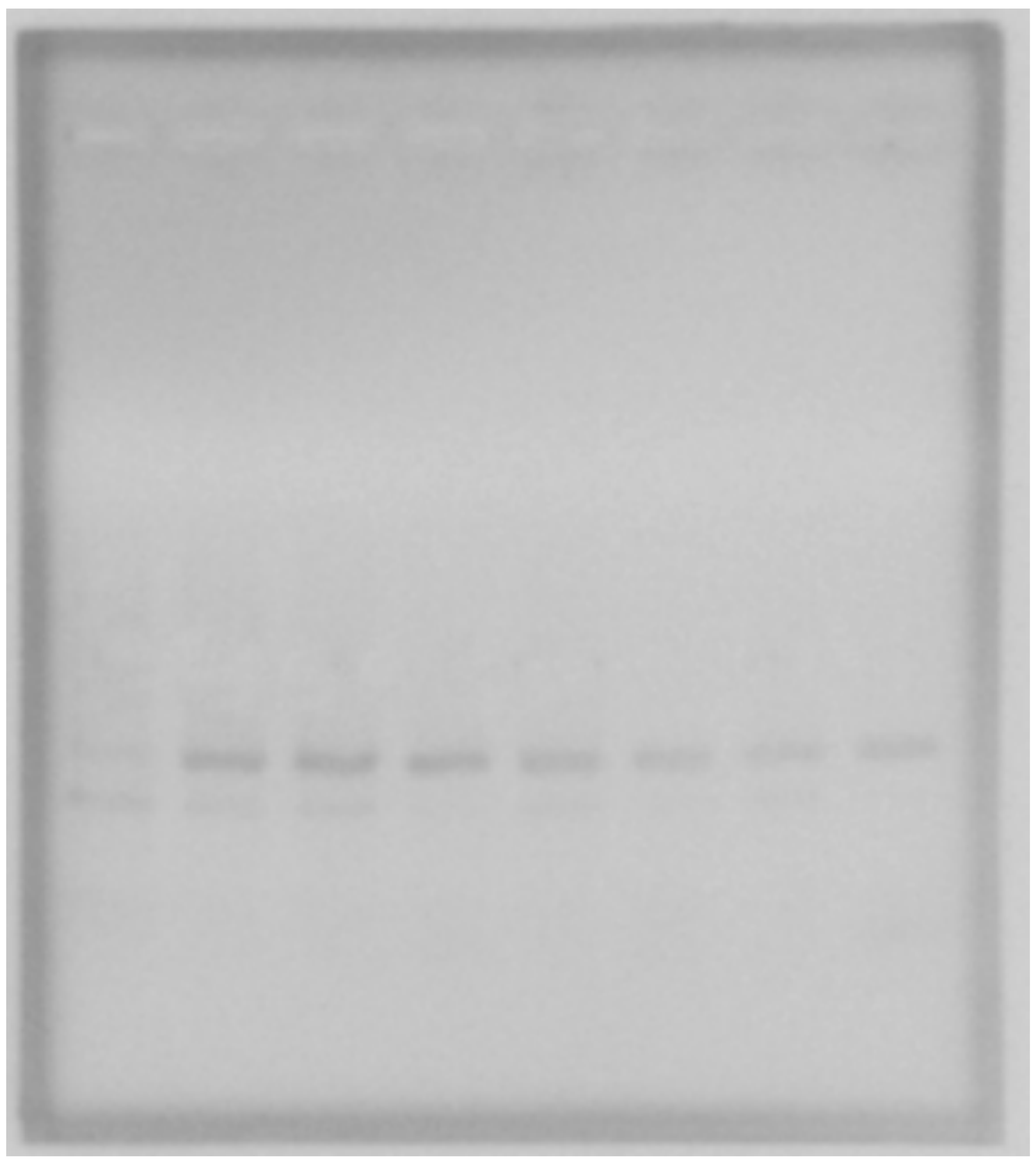

HCFP methanolic extract (µg/mL)	-	-	-	4	6	8	10	12
LPS (1 µg/mL)	-	+	+	+	+	+	+	+
DCF (25 µg/mL)	-	-	+	-	-	-	-	-

TNF-α mRNA expression

HCFP aqueous extract

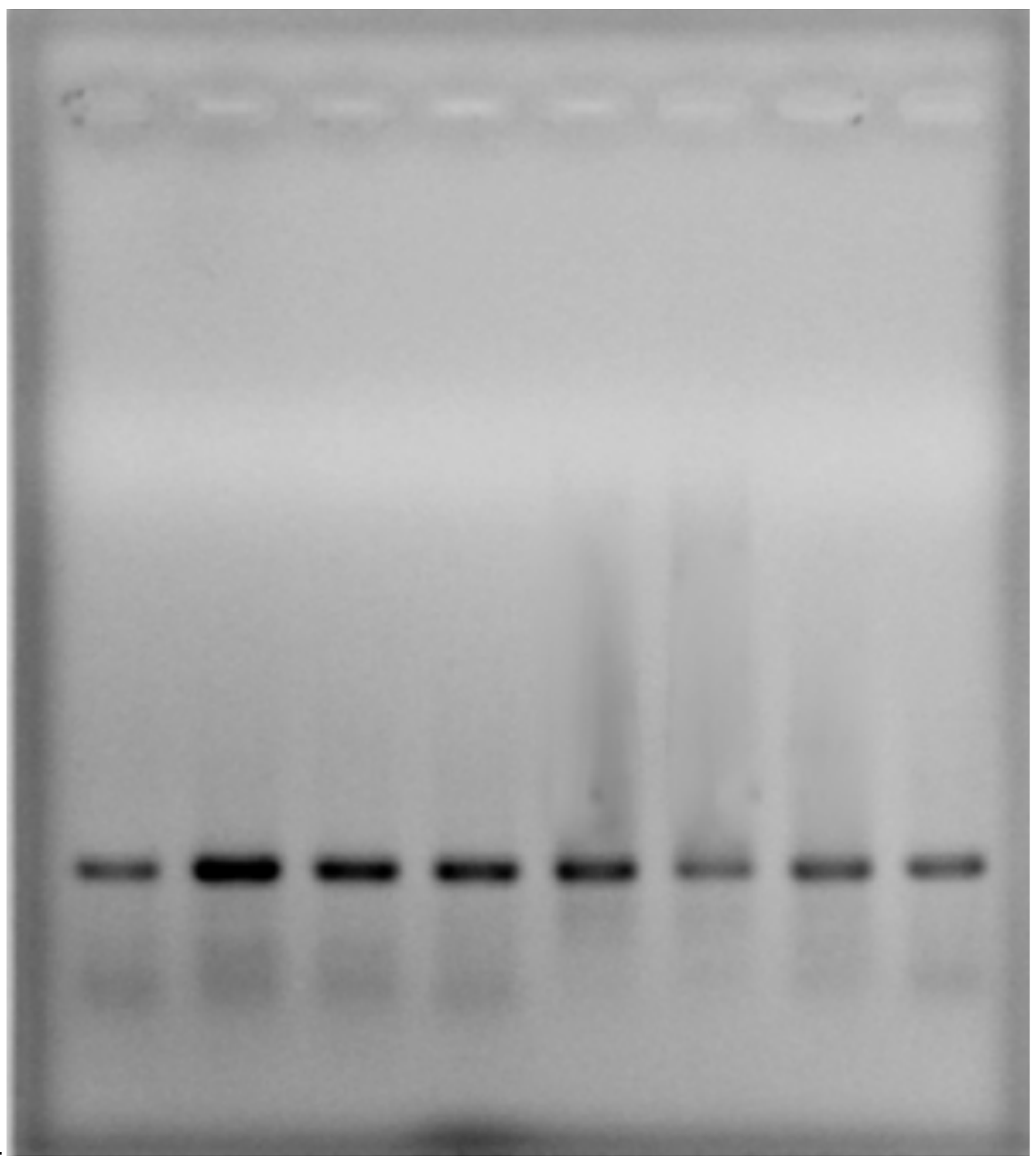

HCFP aqueous extract (µg/mL)	-	-	-	25	50	250	550	750
LPS (1 µg/mL)	-	+	+	+	+	+	+	+
DCF (25 µg/mL)	-	-	+	-	-	-	-	-

HCFP methanolic extract

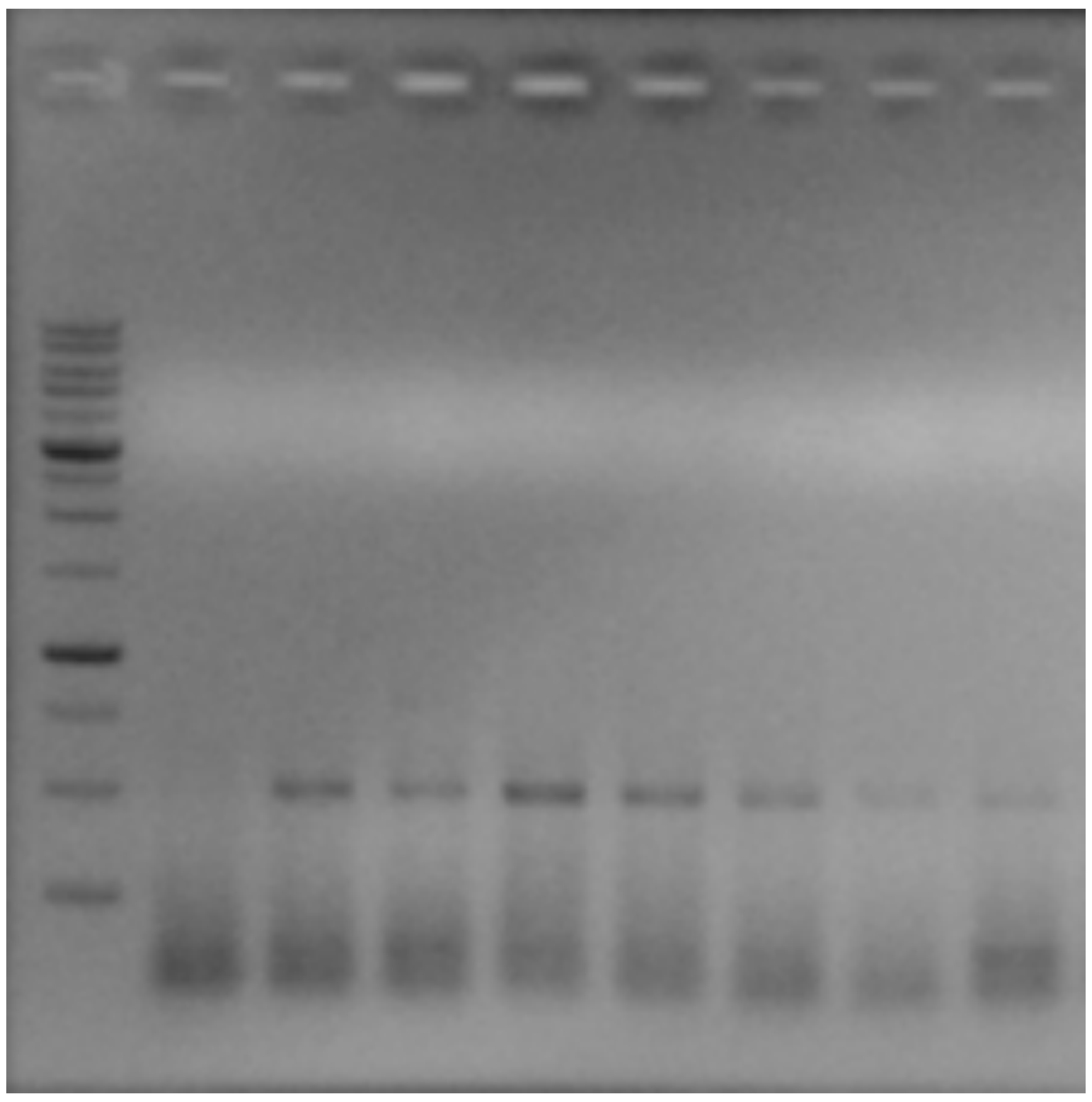

HCFP methanolic extract (µg/mL)	-	-	-	4	6	8	10	12
LPS (1 µg/mL)	-	+	+	+	+	+	+	+
DCF (25 µg/mL)	-	-	+	-	-	-	-	-

IL-6 mRNA expression

HCFP aqueous extract

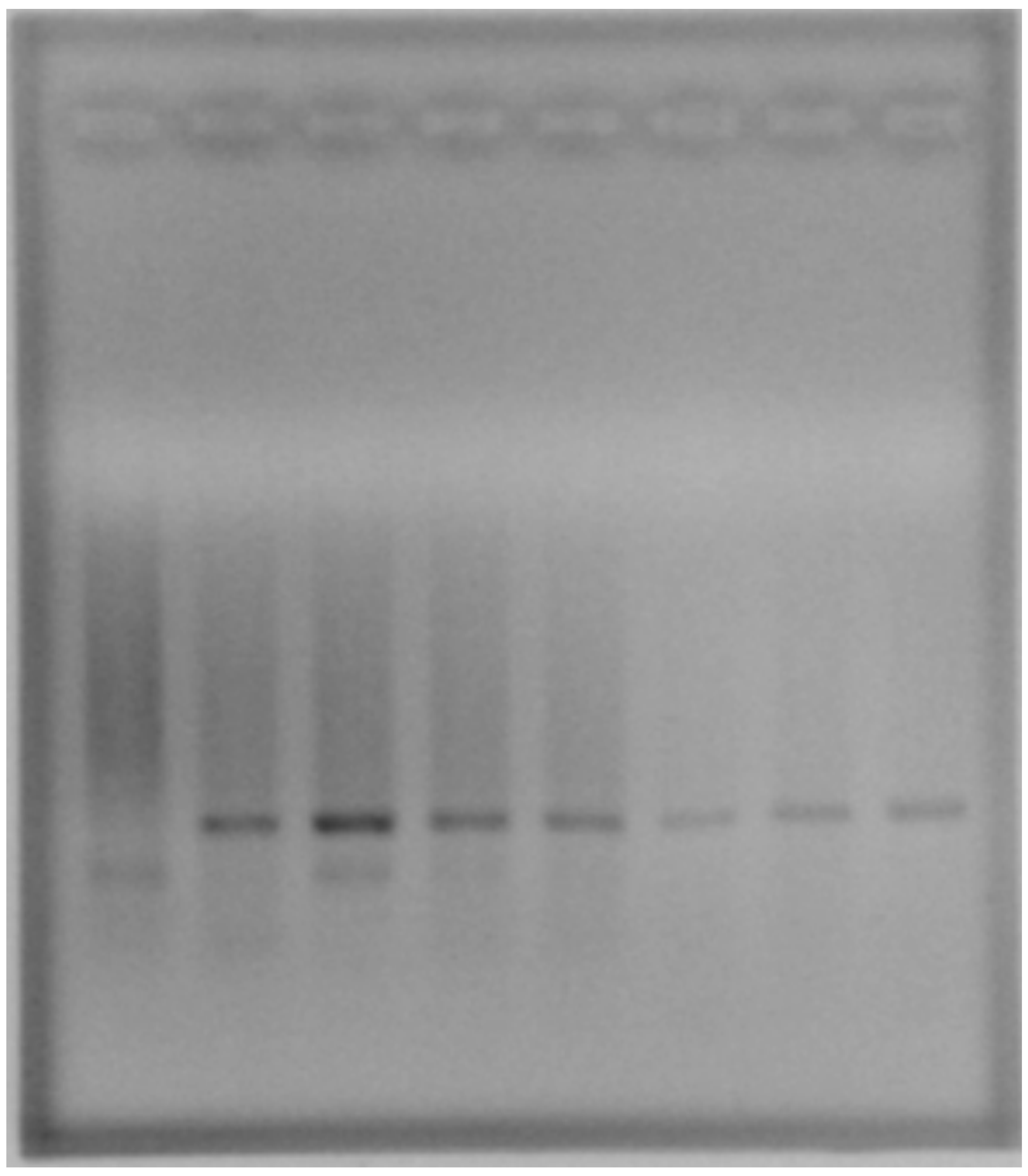

**HCFP aqueous extract (µg/mL)**	-	-	-	**25**	**50**	**250**	**550**	**750**
**LPS (1 µg/mL)**	-	+	+	+	+	+	+	+
**DCF (25 µg/mL)**	-	-	+	-	-	-	-	-

HCFP methanolic extract

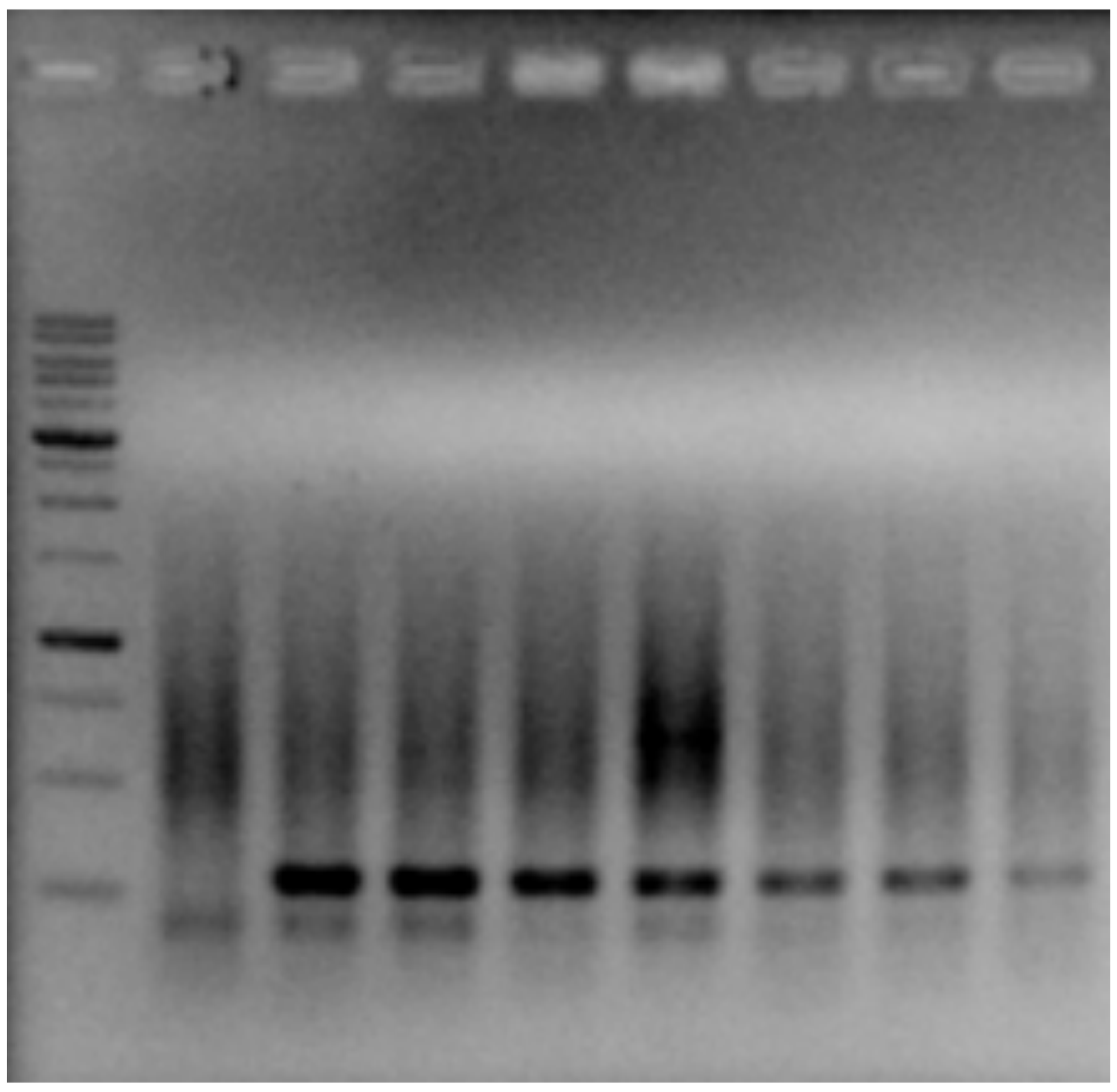

**HCFP methanolic extract (µg/mL)**	-	-	-	**4**	**6**	**8**	**10**	**12**
**LPS (1 µg/mL)**	-	+	+	+	+	+	+	+
**DCF (25 µg/mL)**	-	-	+	-	-	-	-	-

β-Actin mRNA expression

HCFP methanolic extract HCFP aqueous extract

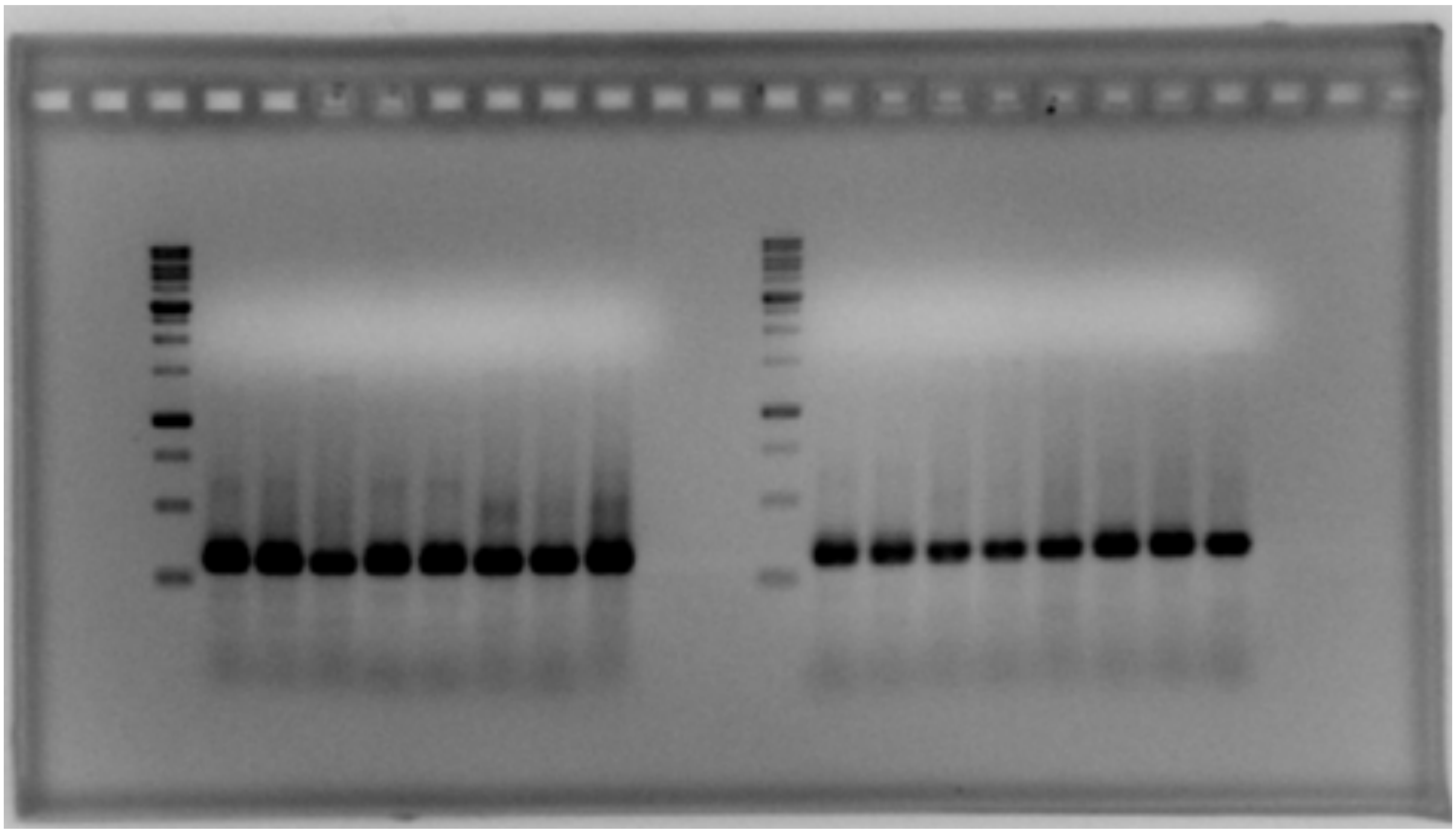

**HCFP methanolic extract (µg/mL)**	-	-	-	**4**	**6**	**8**	**10**	**12**
**LPS (1 µg/mL)**	-	+	+	+	+	+	+	+
**DCF (25 µg/mL)**	-	-	+	-	-	-	-	-

**HCFP aqueous extract (µg/mL)**	-	-	-	**25**	**50**	**250**	**550**	**750**
**LPS (1 µg/mL)**	-	+	+	+	+	+	+	+
**DCF (25 µg/mL)**	-	-	+	-	-	-	-	-

The results of protein expression were visualized using ECL detection reagent and exposed by X-ray film.

Protein expression

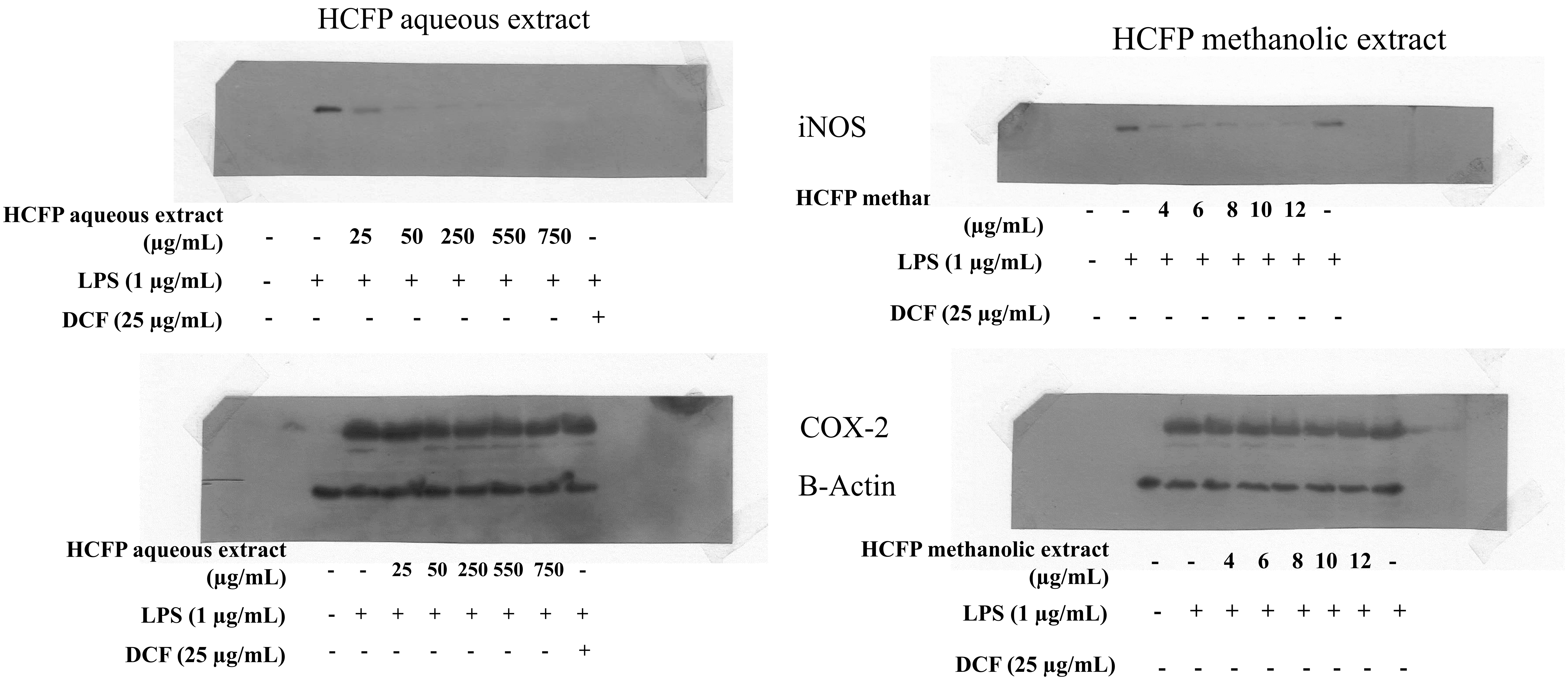

Supplement: S1 Raw images — (PDF) [file pone.0230645.s002.pdf]
