# Supplementary material for: Anti-inflammatory activity of the dietary supplement Houttuynia cordata fermentation product in RAW264.7 cells and Wistar rats
Source: PLoS One. 2020 Mar 25;15(3):e0230645. doi: 10.1371/journal.pone.0230645 (PMC7094840; doi:10.1371/journal.pone.0230645)
Supplement: Attachment — Submitted filename: Response to reviewers.docx [file pone.0230645.s003.docx]

January 31, 2020

PLOS ONE Journal

Dear Dr. Joerg Heber, Editor-in-Chief:

I am enclosing here with a revised manuscript entitled “**Anti-inflammatory activity of the dietary supplement *Houttuynia cordata* fermentation product in RAW264.7 cells and Wistar rats**” for consideration for possible publication as a research paper in the PLOS ONE journal.

As such this paper should be of interest to a broad readership including those interested in a safe and natural source of anti-inflammatory agents, which would be beneficial for alternative therapy and/or dietary prevention of inflammatory diseases.

We have revised the manuscript to address all the concerns from the referees and highlighted all changes in red letters for a revised manuscript.

The list of changes is accompanied with a point-by-point response to all the comments made by the referees.

With the submission of this manuscript I would like to undertake that all of authors have no relation with Prolac (Thailand) Co., Ltd., who is a co-funder with Thailand Research Fund (TRF) on the Research and Researcher for Industry (RRi) project. The company provided 60,000 baht in cash and the HCFP samples (product Lot no. 14/5/2015), while the TRF provided a Ph.D. scholarship and major funding source for the project. This does not alter our adherence to PLOS ONE policies on sharing data and materials. The funders had no role in study design, data collection and analysis, decision to publish, or preparation of the manuscript. However, the funders have been informed and agreed on publishing a manuscript, sharing data and materials.

Thank you for your consideration of this manuscript. Please address all correspondence concerning this manuscript to me and feel free to correspond with me by e-mail.

Sincerely,

Thanaset Senawong, Ph.D.

Email: [sthanaset@kku.ac.th](mailto:sthanaset@kku.ac.th)

**The response to the referees is as following**:

**Journal Requirements:**

When submitting your revision, we need you to address these additional requirements.

1) Please ensure that your manuscript meets PLOS ONE's style requirements, including those for file naming. The PLOS ONE style templates can be found at

http://www.journals.plos.org/plosone/s/file?id=wjVg/PLOSOne_formatting_sample_main_body.pdf and http://www.journals.plos.org/plosone/s/file?id=ba62/PLOSOne_formatting_sample_title_authors_affiliations.pdf

Response: We have checked to ensure that our manuscript meets PLOS ONE's style requirements.

2) At this time, we request that you please report additional details in your Methods section regarding animal care, as per our editorial guidelines:

a) Please provide details of animal (e.g., shelter, food, water, environmental enrichment)

Response: We have provided details of animal regarding shelter, food, water, environmental enrichment in the Cell Culture and Animals section of Materials and Methods (pages 6-7, lines 128-137).

b) please describe any steps taken to minimize animal suffering and distress, such as by administering analgesics,

Response: The researchers have trained for work with animal before doing the research. During conducting the experiment, the researcher worked with animal gently and quickly to reduce pain or suffering of animal. In this study, we did not use the analgesics for animal as we worked with animals in USDA class C pain level. The animal may suffer for a while with no need to administer analgesics.

c) please include the method of sacrifice and

Response: We have added the method of sacrifice in the *in vivo* experiment section of Materials and Methods (page 12, lines 250-252).

d) Please describe the frequency of monitoring and the criteria used to assess animal health and well-being.

Response: The animal health was checked and monitored in detail by the veterinarian of the Northeast Laboratory Center, Khon Kaen University, every day. The criteria used to assess animal health and well-being included appearance of eyes, noses, mouth, anus, and social activity.

3) We also ask that you please provide in your Methods section, additional details regarding the source of the *Houttuynia cordata* fermentation product used in this study. Please provide the product number, lot number, and full ingredient list to ensure reproducibility of the analyses.

Response: We have added additional details as …“ The information on plant ingredient and serving suggestion of the HCFP were obtained from the label on its container. The major ingredients of this HCFP are composed of 99.3% (w/w) aerial parts of *H. cordata* and 0.7 % (w/w) sugar cane powder. Serving suggestion is as follows: 5-15 ml twice a day in the morning before bedtime and before meal. *H. cordata* was cultivated by the Prolac (Thailand) Co., Ltd. in an organic farm in Chai Badan district, Lopburi province, Thailand. The fermentation product Lot no. 14/5/2015 was used throughout the study.”….in Materials section of Materials and Methods (pages 5-6, lines 107-113).

4) Please also clarify whether your animal ethics committee specifically approved this study.

Response: We have mentioned that the approval number was IACUC-KKU-101/60 in Cell Culture and Animals section of Materials and Methods (page 7, line 137). Here is the approval certificate from animal ethics committee who specifically approved this study.

5) PLOS ONE now requires that authors provide the original uncropped and unadjusted images underlying all blot or gel results reported in a submission’s figures or Supporting Information files. This policy and the journal’s other requirements for blot/gel reporting and figure preparation are described in detail at https://journals.plos.org/plosone/s/figures#loc-blot-and-gel-reporting-requirements and https://journals.plos.org/plosone/s/figures#loc-preparing-figures-from-image-files. When you submit your revised manuscript, please ensure that your figures adhere fully to these guidelines and provide the original underlying images for all blot or gel data reported in your submission. See the following link for instructions on providing the original image data: https://journals.plos.org/plosone/s/figures#loc-original-images-for-blots-and-gels.

In your cover letter, please note whether your blot/gel image data are in Supporting Information or posted at a public data repository, provide the repository URL if relevant, and provide specific details as to which raw blot/gel images, if any, are not available. Email us at plosone@plos.org if you have any questions.

Response: The original underlying images are in Supporting Information as “S1_Raw_Images”.

6) Thank you for stating the following financial disclosure:

[This study was supported by the Research and Researcher for Industry (RRi) project, Thailand Research Fund (TRF), which was cooperated with the Prolac (Thailand) Co., Ltd., Lamphun Province, Thailand.].

Please state what role the funders took in the study. If the funders had no role, please state: “The funders had no role in study design, data collection and analysis, decision to publish, or preparation of the manuscript.”

If this statement is not correct you must amend it as needed.

Please include this amended Role of Funder statement in your cover letter; we will change the online submission form on your behalf.

Response: We have added the sentence “The funders had no role in study design, data collection and analysis, decision to publish, or preparation of the manuscript” in our cover letter above and in Acknowledgements section of the revised manuscript (pages 27-28, lines 546-547).

7) Thank you for stating the following in the Competing Interests section:

[The authors have declared that no competing interests exist.].

We note that you received funding from a commercial source: Prolac (Thailand) Co., Ltd

Please provide an amended Competing Interests Statement that explicitly states this commercial funder, along with any other relevant declarations relating to employment, consultancy, patents, products in development, marketed products, etc.

Within this Competing Interests Statement, please confirm that this does not alter your adherence to all PLOS ONE policies on sharing data and materials by including the following statement: "This does not alter our adherence to PLOS ONE policies on sharing data and materials.” (as detailed online in our guide for authors http://journals.plos.org/plosone/s/competing-interests). If there are restrictions on sharing of data and/or materials, please state these. Please note that we cannot proceed with consideration of your article until this information has been declared.

Please include your amended Competing Interests Statement within your cover letter. We will change the online submission form on your behalf.

Please know it is PLOS ONE policy for corresponding authors to declare, on behalf of all authors, all potential competing interests for the purposes of transparency. PLOS defines a competing interest as anything that interferes with, or could reasonably be perceived as interfering with, the full and objective presentation, peer review, editorial decision-making, or publication of research or non-research articles submitted to one of the journals. Competing interests can be financial or non-financial, professional, or personal. Competing interests can arise in relationship to an organization or another person. Please follow this link to our website for more details on competing interests: http://journals.plos.org/plosone/s/competing-interests

[Note: HTML markup is below. Please do not edit.]

Response: We have included the following amended Competing Interests Statement within our cover letter above.

“With the submission of this manuscript I would like to undertake that all of authors have no relation with the Prolac (Thailand) Co., Ltd., who is a co-funder with Thailand Research Fund (TRF) on the Research and Researcher for Industry (RRi) project. The company provided 60,000 baht in cash and the HCFP samples (product Lot no. 14/5/2015), while the TRF provided a Ph.D. scholarship and major funding source for the project. This does not alter our adherence to PLOS ONE policies on sharing data and materials. The funders had no role in study design, data collection and analysis, decision to publish, or preparation of the manuscript. However, the funders have been informed and agreed on publishing a manuscript, sharing data and materials.”

**Reviewers' comments:**

Reviewer's Responses to Questions

Comments to the Author

1. Is the manuscript technically sound, and do the data support the conclusions?

The manuscript must describe a technically sound piece of scientific research with data that supports the conclusions. Experiments must have been conducted rigorously, with appropriate controls, replication, and sample sizes. The conclusions must be drawn appropriately based on the data presented.

Reviewer #1: Partly

Reviewer #2: Yes

Reviewer #3: Yes

Reviewer #4: Yes

2. Has the statistical analysis been performed appropriately and rigorously?

Reviewer #1: Yes

Reviewer #2: Yes

Reviewer #3: Yes

Reviewer #4: Yes

3. Have the authors made all data underlying the findings in their manuscript fully available?

The PLOS Data policy requires authors to make all data underlying the findings described in their manuscript fully available without restriction, with rare exception (please refer to the Data Availability Statement in the manuscript PDF file). The data should be provided as part of the manuscript or its supporting information, or deposited to a public repository. For example, in addition to summary statistics, the data points behind means, medians and variance measures should be available. If there are restrictions on publicly sharing data—e.g. participant privacy or use of data from a third party—those must be specified.

Reviewer #1: No

Reviewer #2: Yes

Reviewer #3: Yes

Reviewer #4: Yes

4. Is the manuscript presented in an intelligible fashion and written in standard English?

PLOS ONE does not copyedit accepted manuscripts, so the language in submitted articles must be clear, correct, and unambiguous. Any typographical or grammatical errors should be corrected at revision, so please note any specific errors here.

Reviewer #1: No

Reviewer #2: Yes

Reviewer #3: Yes

Reviewer #4: Yes

5. Review Comments to the Author

Please use the space provided to explain your answers to the questions above. You may also include additional comments for the author, including concerns about dual publication, research ethics, or publication ethics. (Please upload your review as an attachment if it exceeds 20,000 characters)

**Reviewer #1:** Title: “Anti-inflammatory activity of the dietary supplement Houttuynia cordatafermentation product in RAW264.7 cells and Wistar rats”

Corresponding Author: Thanaset Senawong

In the paper entitled “Anti-inflammatory activity of the dietary supplement Houttuynia cordatafermentation product in RAW264.7 cells and Wistar rats” by Khanutsanan Woranam et al., had showed *Houttuynia cordata* fermentation acts as an Anti-inflammatory RAW264.7 cells and Wistar rats. Overall, the results of this study are of some values in revealing its role in Anti-inflammatory activity.

Below are some examples of issues in which the authors might want to work on.

Many grammar, format and language mistakes are present throughout the manuscript. Manuscript need to have a review by English editor.

Response: The manuscript has been checked for grammatical errors using Grammarly® program (https://www.grammarly.com/) and has been proofreading by native speaker, Associate Professor Dr. Albert J. Ketterman, Institute of Molecular Biosciences, Mahidol University, Salaya Campus, 73170 Thailand.

.

Material and method:

Nitrite determination was performed by spectophotmetry or any other method if so could you please explain this why spectrofluorimetric was not used as this method is considered as attractive due to its facility availability, high sensitivity and selectivity, low limits of detection and low-cost.

Response: Nitrite determination was performed by spectrophotometry. This method is sensitive and selective color reaction for the determination of nitrite and is popularly used by many researchers. This method has been successfully applied to the determination of trace amounts of nitrite and nitrate in water, soil and pharmaceutical preparations. We have set up this method in our lab due to availability of spectrophotometer instead of fluorospectrometer.

Primer sequences placed in method section should be added as supplementary table.

Response: We have placed the primer sequences to “S1 Table Primer list” in Supporting Information.

Was time course study performed when incubated with LPS (1 μg/mL) in case of RNA isolation and protein extraction.

Response: We have not performed time course study for LPS treatment. We performed 6 h incubation for RNA isolation and 24 h for protein isolation according to our predetermined time course data.

Result section:

How many sets of experiments were performed for the cell viability assay? Could you add cell pictures before and after treatment?

Response: We performed 3-5 independent experiments for the cell viability assay. The cell morphology was also observed under inverted microscope. Here is an example of cell morphology observed under inverted microscope. However, we cannot not put all images because we have not taken all images during performing the experiments although we have monitored all images.

In figure 2C the expression level of IL-1β when treated HCFP methanolic extract

At 12 (μg/mL) showed decreased level of expression while in quantitative results figure 2D showed increased level. Could you please explain which method was used for the quantitative analysis?

Response: We quantitate the band intensity using Quantity One software 4.4.1 (Bio-Rad). We have checked the result and found that the expression level of IL-1β in figure 2C when treated HCFP methanolic extract at 12 μg/mL showed decreased level of expression, however, the quantitative results in figure 2D did not show an increased level as mentioned by the reviewer 1. The quantitative results in figure 2D actually showed decreased level corresponding to figure 2C.

Discussion

The discussion part is poorly described it need to be revised and latest references should be added.

Response: We have thoroughly checked the discussion part for a revised manuscript. Two latest references were added as ref. 32 and 38.

The manuscript is not acceptable in the present format. It needs to be extensively revised.

Response: The revised manuscript has been proofreading by native speaker, Associate Professor Dr. Albert J. Ketterman, Institute of Molecular Biosciences, Mahidol University, Salaya Campus, 73170 Thailand.

Reviewer #2: The manuscript is providing some nice conclusions about a potent dietary supplement, however, I have a concern about the conclusion. This needs to be rewritten according to the results obtained and should't be so general. Further not a single reference is quoted from the literature published in 2019. Hence, the latest references should be added.

Response: Thank you for reviewer’s comments. We have added two latest references as ref. 32 and 38.

Reviewer #3: The paper is very interesting and designed diliginty in a well professional manner. The paper entitled"Anti-inflammatory activity of the dietary supplement Houttuynia cordatafermentation product in RAW264.7 cells and Wistar rats" is considered a new approach in combining effect of *Houttuynia cordata* fermentation product both *in vivo* and *in vitro* by carrying out well designed experiments and performing cell viability test by explanation in-details and the authors reported all the kits sources and also the approval number of animal ethics in their university, the statistical analysis was performed professionally , the discussion part is brilliant in explaining the causes of inflammation and the efficacy of the used extract .

So I recommend publication of this paper which deserve publication and will be of great benefit for readers.

Response: Thank you for reviewer’s comments.

Reviewer #4: Thank you to give me the opportunity to review this paper

I found this is interesting.

My comments:

1. I am warried that the HCFP has a degraded the protein or has cytotoxicity on the cells.

Response: We do not think that HCFP has a degraded protein or has cytotoxicity on the cells because we have observed cell morphology under inverted microscope and no cell death was observed at the concentrations tested.

2. It suggested to do another viability assay to confirm the result

Thanks

Response: The cell morphology was also observed under inverted microscope as mentioned above.
